# Tele-Instruction Tool for Multiple Lay Responders Providing Cardiopulmonary Resuscitation in Telehealth Emergency Dispatch Services: Mixed Methods Study

**DOI:** 10.2196/46092

**Published:** 2023-07-26

**Authors:** Jianing Xu, Mingyu Qu, Xuejie Dong, Yihe Chen, Hongfan Yin, Fangge Qu, Lin Zhang

**Affiliations:** 1 School of Public Health Shanghai Jiao Tong University Shanghai China; 2 Xinhua Hospital Shanghai Jiao Tong University School of Medicine Shanghai China; 3 Department of Global Health School of Public Health Peking University Beijing China; 4 School of Nursing Shanghai Jiao Tong University Shanghai China

**Keywords:** cardiac arrest, lay responder, teamwork, telehealth, telephone-assisted cardiopulmonary resuscitation

## Abstract

**Background:**

Telephone-assisted cardiopulmonary resuscitation (T-CPR) has proven to be a crucial intervention in enhancing the ability of lay responders to perform cardiopulmonary resuscitation (CPR) during telehealth emergency services. While the majority of established T-CPR protocols primarily focus on guiding individual rescuers, there is a lack of emphasis on instructing and coordinating multiple lay responders to perform resuscitation collaboratively.

**Objective:**

This study aimed to develop an innovative team-based tele-instruction tool to efficiently organize and instruct multiple lay responders on the CPR process and to evaluate the effectiveness and feasibility of the tool.

**Methods:**

We used a mixed methods design in this study. We conducted a randomized controlled simulation trial to conduct the quantitative analysis. The intervention groups used the team-based tele-instruction tool for team resuscitation, while the control groups did not have access to the tool. Baseline resuscitation was performed during the initial phase (phase I test). Subsequently, all teams watched a team-based CPR education training video and finished a 3-person practice session with teaching followed by a posttraining test (phase II test). In the qualitative analysis, we randomly selected an individual from each team and 4 experts in emergency medical services to conduct semistructured interviews. The purpose of these interviews was to evaluate the effectiveness and feasibility of this tool.

**Results:**

The team-based tele-instruction tool significantly improved the quality of chest compression in both phase I and phase II tests. The average compression rates were more appropriate in the intervention groups compared to the control groups (median 104.5, IQR 98.8-111.8 min^–1^ vs median 112, IQR 106-120.8 min^–1^; *P*=.04 in phase I and median 117.5, IQR 112.3-125 min^–1^ vs median 111, IQR 105.3-119 min^–1^; *P*=.03 in phase II). In the intervention group, there was a delay in the emergency response time compared to that in the control group (time to first chest compression: median 20, IQR 15-24.8 seconds vs median 25, IQR 20.5-40.3 seconds; *P*=.03; time to open the airway: median 48, IQR 36.3-62 seconds vs median 73.5, IQR 54.5-227.8 seconds; *P*=.01). However, this delay was partially mitigated after the phase II test. The qualitative results confirmed the compatibility and generalizability of the team-based tele-instruction tool, demonstrating its ability to effectively guide multiple lay responders through teamwork and effective communication with telecommunicators.

**Conclusions:**

The use of the team-based tele-instruction tool offers an effective solution to enhance the quality of chest compression among multiple lay responders. This tool facilitated the organization of resuscitation teams by dispatchers and enabled efficient cooperation. Further assessment of the widespread adoption and practical application of the team-based tele-instruction tools in real-life rescue scenarios within the telehealth emergency services system is warranted.

## Introduction

Technological advancements, particularly in telehealth services, have opened up new possibilities for improving the quality and accessibility of health care [1,2]. The latest guidelines from the American Heart Association (AHA) and European Resuscitation Council suggest the implementation of telephone-assisted cardiopulmonary resuscitation (T-CPR) instructions by telecommunicators to assist lay responders (Class 1; Level of Evidence C-LD) [3,4]. In today’s era of widespread mobile devices, telephone interactions play a crucial role in connecting emergency medical telecommunicators with callers at the scene of an incident. Telecommunicators can initially recognize out-of-hospital cardiac arrests (OHCA) and provide step-by-step instructions on how to perform cardiopulmonary resuscitation (CPR) [5]. This approach is recognized as safe and effective in improving CPR quality and patient survival, regardless of whether individuals are trained or untrained [6]. Extensive evidence supports the notion that receiving T-CPR increases the likelihood of a lay responder providing CPR and improves outcomes, such as sustained return of spontaneous circulation and favorable neurological recovery after hospital discharge [7-11].

The existing T-CPR protocols for lay responders primarily focus on single-rescuer CPR and lack guidance on promoting collaboration among multiple lay responders to enhance prearrival care. Studies have shown that 43.1% of OHCA rescue events involve the presence of multiple lay responders at the scene [12]. In our previous study, it was observed that even though multiple lay responders possessed proficiency in single-rescuer CPR, they were unable to achieve optimal teamwork without team educational training [13]. However, there was a notable prevalence of unsatisfactory performance in terms of task allocation, effective communication, and teamwork, which was disappointing. Given the time-consuming and labor-intensive nature of resuscitation, it is crucial to instruct multiple lay responders as a well-coordinated team. By promoting team cooperation, the quality of CPR can be enhanced while alleviating the burden on individual rescuers. Additionally, optimal role distribution within the team can ensure prompt defibrillation [14].

Introducing a team-based tele-instruction plan for telecommunicators could serve as a cost-effective yet highly beneficial strategy to optimize the effectiveness of team cooperation among multiple lay responders. This plan would ensure the consistent delivery of high-quality CPR for patients who experience OHCA. In contrast to periodic mass training initiatives for team-based CPR, implementing the team-based tele-instruction plan through telecommunicators leverages its widespread reach to effectively instruct a large number of lay responders, resulting in enhanced resuscitation quality. Hence, this study aimed to develop an innovative team-based tele-instruction tool with the aim of expanding the use of telehealth emergency services. We evaluated the effectiveness and feasibility of this tool through a randomized controlled simulation trial and semistructured interviews.

## Methods

**Study Design**

We used a mixed methods approach in this study. We designed an innovative team-based tele-instruction tool for lay responders based on updated guidelines and an extensive literature study [3,4,13]. Subsequently, we implemented the team-based tele-instruction tool in a randomized controlled simulation trial. Following the trial, we randomly selected an individual from each team and 4 experts to conduct semistructured interviews.

### Team-Based Tele-Instruction Tool for T-CPR

In cooperation with a Chinese company specializing in dispatch systems, we innovatively designed a team-based tele-instruction protocol and developed a test tool for the medical dispatch system; however, it is currently used only for research purposes. If the response to the question “Can you find other people to help you?” was answered as “Yes,” the system would link to the team-based tele-instruction protocol.

**Figure 1:**
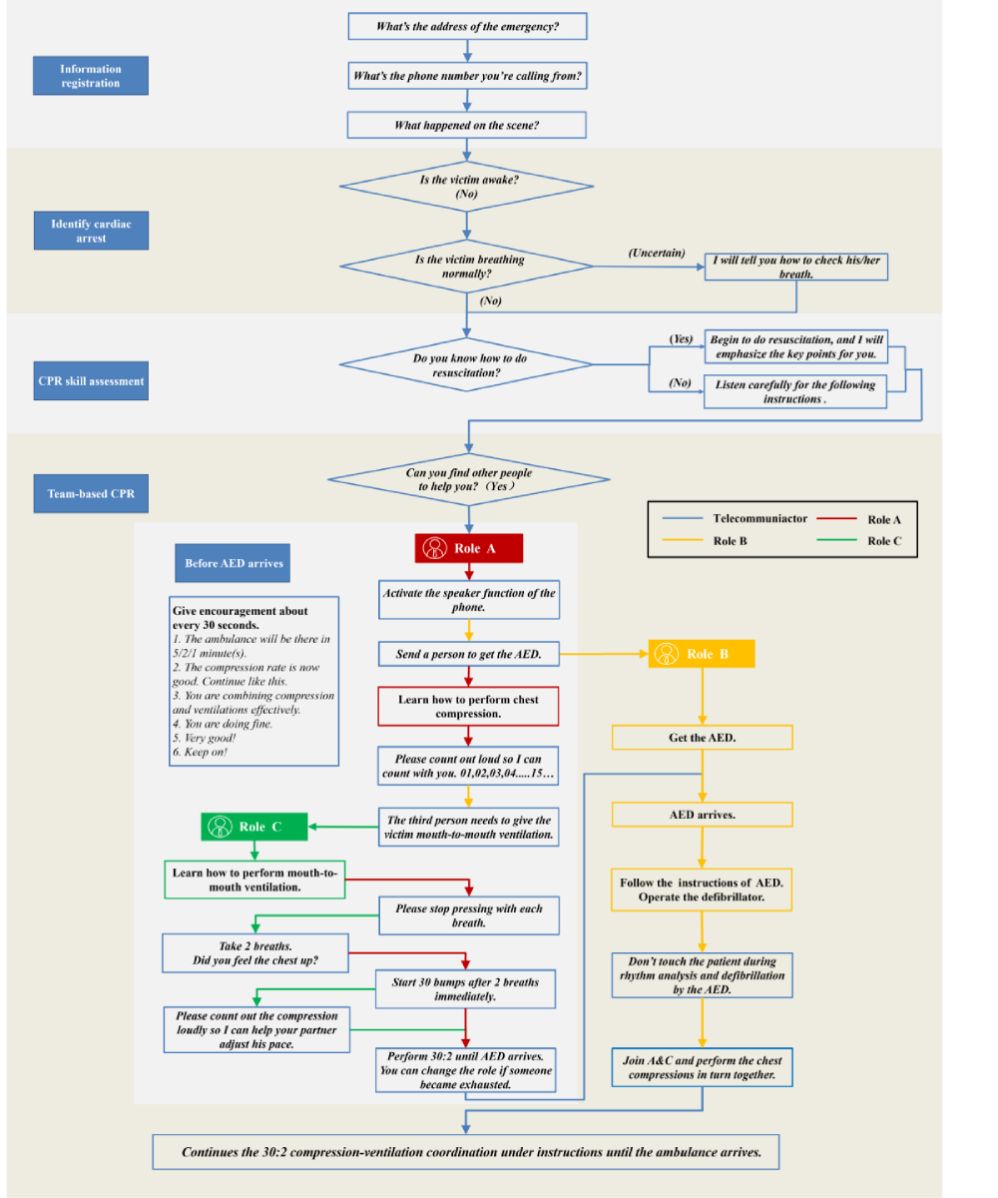
Team-based tele-instruction protocol. Roman font type means performances; italic font type means words. AED: automated external defibrillator; CPR: cardiopulmonary resuscitation.

The protocol was applied to resuscitation teams of three or more lay responders, and the medical emergency response process was divided into 2 parts: pre-CPR instructions and team-based CPR instructions. The pre-CPR instructions included information registration, identification of cardiac arrest, and evaluation of lay responders’ CPR skills. Team-based CPR instructions included chest compressions, mouth-to-mouth ventilation, automated external defibrillator (AED) support, and continuous encouragement (Figure 1). The protocol concisely defined the roles and responsibilities of each member and emphasized the methods and importance of reasonable task allocation, leadership, and closed-loop communication within the resuscitation team.

### Randomized Controlled Simulation Trial

From June 1 to December 31, 2021, 132 participants aged 18-60 years were recruited. Participants who had undergone clinical practice, were engaged in medical or emergency-related occupations, or were physically unable to perform high-intensity testing and training were excluded. To ensure that all participants possessed the necessary skills in single-rescuer CPR, we initially conducted a basic life support training called WeCan CPR. This training encompassed essential skills, such as contacting the emergency dispatch center (dispatch help number: 1-2-0), performing chest compressions, providing mouth-to-mouth ventilation, and using an AED [15].

**Figure 2:**
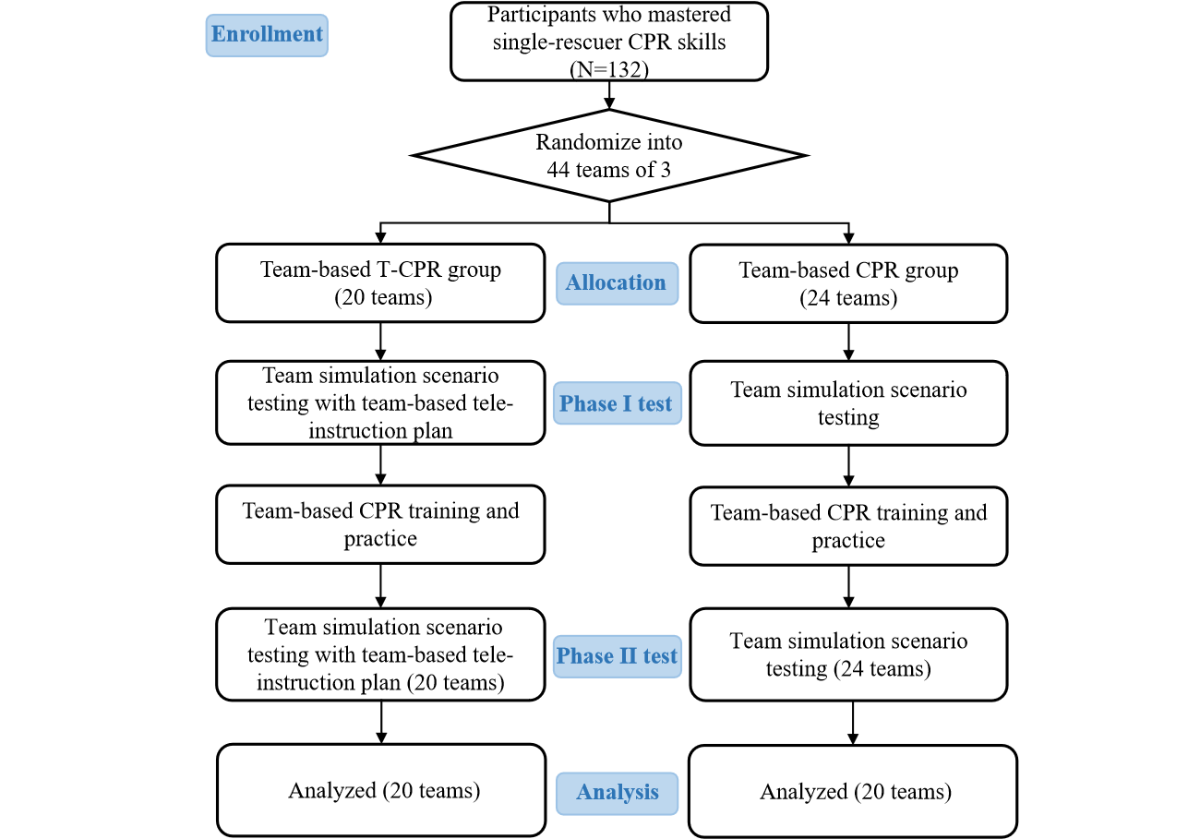
Flow diagram of the study. Phase I tests were team-based cardiopulmonary resuscitation (CPR) performance and capabilities assessment with or without the team-based tele-instruction tool among individuals who have undergone single-rescuer CPR training. Phase II tests were team-based CPR performance and capabilities assessment with or without the team-based tele-instruction tool among individuals who have undergone single-rescuer and team-based CPR training. T-CPR: telephone-assisted cardiopulmonary resuscitation.

Subsequently, participants who passed the single-rescue CPR skill test were randomized into teams of 3. Each team was randomly assigned to 1 of the 2 study groups: the control group and the intervention group. In the control group, participants did not receive any guidance from a telecommunicator. In contrast, in the intervention group, one of the researchers (LZ) instructed the participants to use the team-based tele-instruction tool. Each team was asked to perform resuscitation before the team-based CPR training (phase I test) at baseline. Following the phase I test, each team underwent a 30-minute team training separately, following the same protocol as our previous study [13]. The team-based CPR educational training comprised a 10-minute real-person demonstration video and a practice session with teaching in groups of 3 (20 minutes). The content of the video module strictly referenced the team-based CPR protocol, which highlighted the critical elements of communication skills (call-out and check-back), cooperation skills (mutual support and knowledge sharing), and leadership skills (maintenance of a global perspective and task management). The teaching practice was conducted under the guidance of our researchers (XD and YC) until each team was proficient in the skills. Subsequently, each team underwent a phase II test with the same cardiac arrest simulation scenario as that during the first test (Figure 2).

In the team-based CPR simulated scenario, each team was required to assign and finish tasks, including contacting the emergency telecommunicator, performing chest compressions, mouth-to-mouth ventilation, and retrieving and using an AED. In the simulated scenario, the telecommunicator was blinded to the team’s CPR performance and activated the medical emergency response as soon as the call went through from the team-based T-CPR group. The scenario commenced as each team entered the room and concluded 10 minutes after they initiated the first chest compression. Participant B left the room to retrieve the AED (Laerdal AED Trainer 2, Laerdal Medical) and was instructed to return to the room after 5 minutes.

No feedback was provided to the participants once the scenario began. Quantitative data were collected from manikin feedback (Resusci Anne QCPR, Laerdal Medical), video recordings, and the evaluations of researchers and submitted to a blinded assessor. A total of 3 cameras captured the algorithm time data, CPR technique, and communication with the telecommunicator from different angles (head, side, and above). The quality of chest compressions and ventilation was recorded using SimPad PLUS (Laerdal Medical). The AED performance was assessed by researchers using a dichotomous (yes or no) format. Time intervals, including hands-off time and a delay at the beginning, were measured using a stopwatch. The participants’ age, sex, and self-reported body weight were also documented.

### Qualitative Semistructured Interviews

Semistructured interviews were conducted to evaluate the effectiveness and feasibility of the tool from the perspectives of telecommunicators and lay responders.

#### Participants’ Interview

Semistructured interviews were conducted after the phase II test to explore the acceptability of the protocol. We selected 1 individual from each T-CPR team using a random number table. All interviews included open-ended questions to elicit detailed accounts of the participants’ experiences with the protocol, with subsequent probing questions based on the interviewees’ responses. The questions were divided into 2 dimensions: participants’ attitudes toward teamwork and their interactions with the telecommunicator. A total of 5 themes were identified, including operational skills, team cooperation, communication with the telecommunicator, team-based CPR instructions, and other opinions.

The interviews began with a question about the individual’s role in the simulation, followed by seeking their viewpoints on instructions provided by telecommunicators. Questions were specifically tailored to teamwork and leadership during resuscitation and quality efforts in CPR and other areas. Lastly, we inquired about the overall processes related to the tool, their views about this tool’s best practices, and the areas where they felt improvement was most needed. With the permission of the participants, each interview was audio-recorded and fully transcribed by independent professional transcriptionists within 24 hours of each interview and reviewed by the interviewers for accuracy. Data analysis was performed concurrently with data collection.

#### Expert Consultation

Expert consultations were conducted to enhance the clarity and efficiency of instructing the caller on the required actions and procedures while also ensuring that the protocol remained scientifically sound, logical, and feasible. Interviews were conducted to address 3 aspects of the protocol: interior logic verification, exterior contrast analysis, and generalizability analysis. The 4 experts involved in the study were experienced specialists in the fields of emergency medical services and T-CPR dispatch protocol. They can play a substantial role in supervising the team-based T-CPR instruction protocol and participating in the development of the tool promotion plan in China.

### Statistical Analysis

Quantitative data are presented as frequencies with percentages for categorical variables and median (IQR) for continuous variables. Differences in categorical outcomes were assessed using the chi-square test, and continuous variables were compared between the 2 groups using the Wilcoxon rank sum test. All statistical analyses were performed using SPSS version 24.0 (IBM Corp). Two-sided *P* values <.05 were considered statistically significant.

A qualitative analysis was conducted by 2 researchers using Colaizzi’s phenomenological method [16]. Researchers read the set of transcripts for each case in their entirety, identifying significant phrases, followed by line-by-line coding and the organization of codes into larger categories at each point in time. To ensure a comprehensive understanding of the transcripts, 2 researchers independently reviewed them before arriving at a consensus, and 3 study participants were invited to review the exhaustive statement. Through constant comparison, the data achieved saturation in our coding scheme by the 13th transcript, although we analyzed all 20 transcripts.

### Ethics Approval

Ethical approval was obtained from the Joint Research Ethics Board of the Shanghai Jiao Tong University School of Public Health and Nursing (SJUPN-201913). All participants were verbally informed about the intentions of the study, and they provided written informed consent. To protect the privacy of the participants, the data were anonymized by assigning participant IDs to individuals for representation. Our team provided free basic life support training and a 50 RMB (approximately US $8) present to each participant upon completion of the study to compensate for their time and feedback.

## Results

### Results of the Randomized Controlled Simulation Trial

A total of 132 participants were randomly divided into 44 teams of 3 members each; of these, 48 (36.4%) were male. The age of the participants ranged from 18 to 60 years, with a median age of 20 (IQR 18-30) years in the team-based T-CPR group and 19 (IQR 18-30) years in the team-based CPR group. The mean weight was 63.6 (SD 12.7) kg and 63.6 (SD 14.9) kg in the team-based T-CPR and team-based CPR groups, respectively. The demographic characteristics of the 2 groups were not significantly different.

**Figure 3:**
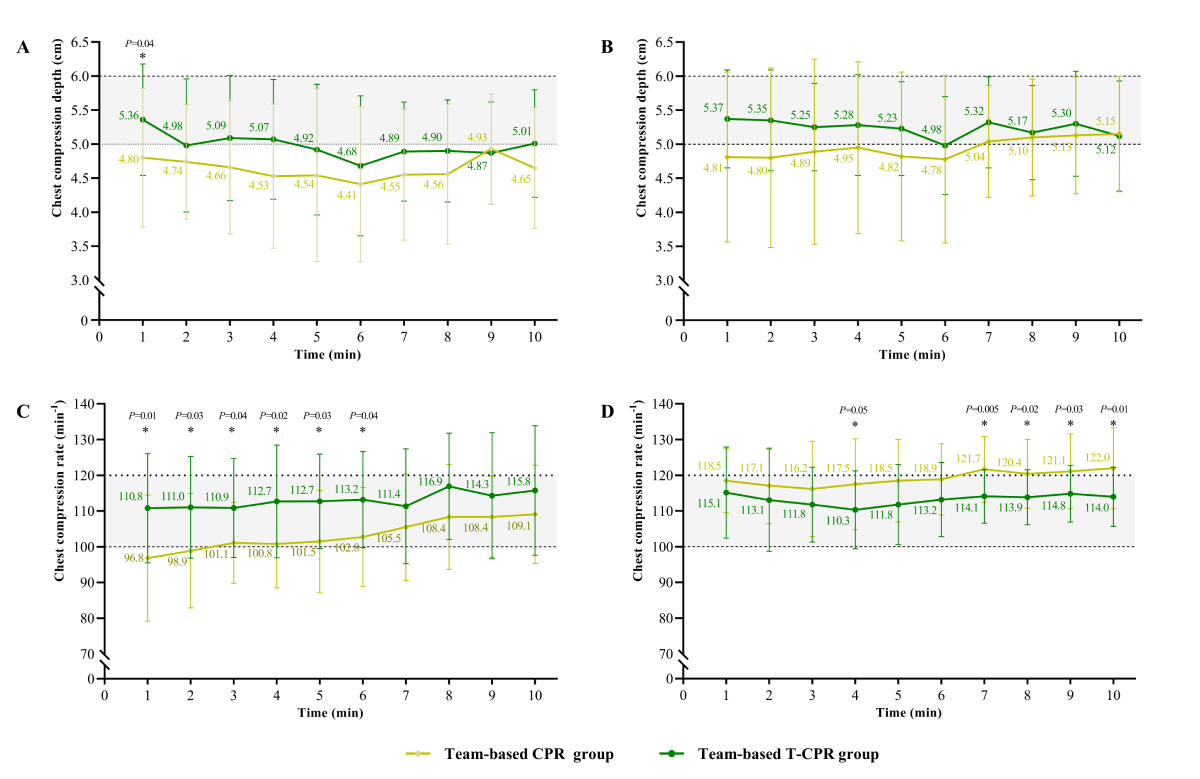
Trends of chest compression depth and rate between 2 groups. (A) Trends of chest compression depth in phase I test. (B) Trends of chest compression depth in phase II test. (C) Trends of chest compression rate in phase I test. (D) Trends of chest compression rate in phase II test. Phase I tests were team-based CPR performance and capabilities assessment with or without the team-based tele-instruction tool among individuals who have undergone single-rescuer CPR training. Phase II tests were team-based CPR performance and capabilities assessment with or without the team-based tele-instruction tool among individuals who have undergone single-rescuer and team-based CPR training. CPR: cardiopulmonary resuscitation; T-CPR: telephone-assisted cardiopulmonary resuscitation.

This protocol was conducive to the quality of CPR, especially for maintaining an appropriate compression frequency. The intervention group demonstrated higher adherence to the guideline for average compression rates (100-120 min^–1^) in both phase I and II tests (median 104.5, IQR 98.8-111.8 min^–1^ vs median 112, IQR 106-120.8 min^–1^; *P*=.04; and median 117.5, IQR 112.3-125 min^–1^ vs median 111, IQR 105.3-119 min^–1^; *P*=.03, respectively) (Table 1). The telecommunicator’s instructions regarding role assignment and effective cooperation resulted in a significantly higher chest compression rate in the first 6 minutes of the phase I test (Figure 3C). This phenomenon was also observed after training when the transition of the chest compression implementer was completed by the sixth minute (Figure 3D).

The compression depth of the intervention group was significantly greater in the first minute of the phase I test (Figure 3A), and the depth was relatively greater in the intervention group almost at all times (Figures 3A and 3B). The percentage of performing 30 compression cycles and 2 resuscitations significantly improved with the protocol in the phase II test (n=11, 45.8% vs n=16, 80%; *P*=.02). The percentage of clearing, while the AED delivered shock in the phase I test, was significantly higher in the intervention group (n=2, 8.7% vs n=8, 40%; *P*=.03).

In the phase I test, the emergency response time of the team-based T-CPR group was significantly longer than that of the team-based CPR group (Table 2), including time to first chest compression (median 20, IQR 15-24.8 seconds vs median 25, IQR 20.5-40.3 seconds; *P*=.03), time to open the airway (median 48, IQR 36.3-62 seconds vs median 73.5, IQR 54.5-227.8 seconds; *P*=.01), and the time to the first shock (median 77, IQR 66-93 seconds vs median 105.5, IQR 89.3-175 seconds; *P*<.001). Training partially reduced the delay in emergency response time, as indicated in the phase II test, where the difference in the time to first chest compression was not statistically significant compared with the time to open the airway (median 43.5, IQR 37.3-50.8 seconds vs median 73.5, IQR 41.8-71.3 seconds; *P*=.03) and the time to the first shock (median 80, IQR 66-90 seconds vs median 94, IQR 81.8-115.8 seconds; *P*=.01).

**Table 1:** Cardiopulmonary resuscitation (CPR) operation assessment in the team-based CPR simulation scenario. Phase I tests were team-based CPR performance and capabilities assessment with or without the team-based tele-instruction tool among individuals who have undergone single-rescuer CPR training. Phase II tests were team-based CPR performance and capabilities assessment with or without the team-based tele-instruction tool among individuals who have undergone single-rescuer and team-based CPR training.

Parameters			Phase Ⅰ							Phase Ⅱ				
			Intervention group (n=20)		Control group (n=24)		*P* value^a^		Intervention group (n=20)			Control group (n=24)		*P* value^a^
**Overall**														
	CCF^b^, n (%)	61.5 (52-67.5)		61.5 (54.5-75.5)		.72		67.5 (58.3-77)			64.5 (58.3-74)		.64	
	Hands-off time (seconds), median (IQR)	218.3 (192.9-288.4)		237.8 (143.9-281)		.99		219.3 (153.3-252.1)			207.9 (149-250.1)		.82	
	Overall score (percentage, 100 in total), median (IQR)	24.5 (17-44.5)		24 (7-38.8)		.60		42 (16.3-67.3)			35.5 (14.3-58.5)		.39	
**Chest compression**														
	Average compression depth (cm), median (IQR)	5.3 (4.4-5.6)		4.7 (4.2-5.2)		.15		5.3 (4.9-5.8)			5.2 (4.5-5.5)		.25	
	Deep enough compression (5-6 cm), n (%)^c^	7 (35)		12 (50)		.37		9 (52.9)			15 (62.5)		.75	
	Average compression rate (min^−1^), median (IQR)	112 (106-120.8)		104.5 (98.8-111.8)		.04		111 (105.3-119)			117.5 (112.3-125)		.03	
	Compression with adequate rate (100-120 min^–1^), n (%)^c^	15 (75)		15 (62.5)		.52		13 (76.5)			14 (58.3)		.32	
	Compression score (percentage, 100 in total), median (IQR)	58 (38-66.5)		54 (34.3-80)		.61		78.5 (63.5-85)			74.5 (62.8-86.3)		.80	
**Ventilation**														
	Average ventilation volume (ml), median (IQR)	523 (458.3-590)		495.5 (0-617.8)		.47		633.5 (481-702)			516.5 (94.8-658)		.06	
	Perform circles of 30 compressions and 2 breaths, n (%)^c^	13 (65)		11 (45.8)		.20		16 (80)			11 (45.8)		.02	
	Ventilation score (percentage, 100 in total), median (IQR)	47 (22.3-66)		52.5 (0-68)		.97		52.5 (24.3-70.5)			50 (3-73.5)		.61	
**AED^d^ support**														
	Keep compression without flow time after AED arrives, n (%)^c^	10 (50)		15 (62.5)		.54		16 (94.1)			18 (75)		.21	
	Clear while AED delivers the shock, n (%)^c^	8 (40)		2 (8.7)		.03		9 (52.9)			9 (39.1)		.52	

^a^*P* values were derived by Wilcoxon rank sum test.

^b^CCF: chest compression fraction.

^c^*P* values were derived by chi-square test.

^d^AED: automated external defibrillator.

**Table 2:** Participants’ emergency response time assessment in the team-based cardiopulmonary resuscitation (CPR) simulation scenario. Phase I tests were team-based CPR performance and capabilities assessment with or without the team-based tele-instruction tool among individuals who have undergone single-rescuer CPR training. Phase II tests were team-based CPR performance and capabilities assessment with or without the team-based tele-instruction tool among individuals who have undergone single-rescuer and team-based CPR training. The emergency medical number in mainland China is 1-2-0.

Time intervals (in seconds)	Phase Ⅰ				Phase Ⅱ		
	Intervention group (n=20), median (IQR)	Control group (n=24), median (IQR)	*P* value^a^	Intervention group (n=20), median (IQR)		Control group (n=24), median (IQR)	*P* value^a^
Time to cardiac arrest identification	6.5 (4.3-16.5)	9 (7-11)	.36	5 (4-10)		6.5 (4.3-8.8)	.55
Time to call emergency dispatch number	14.5 (7.3-26)	13 (10-17.5)	.68	19.5 (8.3-21.8)		13.5 (9-20)	.24
Time to first chest compression	25 (20.5-40.3)	20 (15-24.8)	.03	19.5 (11.3-27.5)		18.5 (12.5-21.8)	.65
Time to open the airway	73.5 (54.5-227.8)	48 (36.3-62)	.01	58 (41.8-71.3)		43.5 (37.3-50.8)	.03
Time to first shock^b^	105.5 (89.3-175)	77 (66-93)	<.001	94 (81.8-115.8)		80 (66-90)	.01
Time to resume CPR after shock	8 (3.5-12)	4.5(3-12)	.57	4.5 (3-6)		3.5 (2-4.5)	.14

^a^*P* values were derived by Wilcoxon rank sum test.

^b^Including 5 minutes spent for the retrieval of automated external defibrillator.

### Results of the Qualitative Semistructured Interviews

When inquired about teamwork, most responses were positive, particularly regarding team cooperation.

When I felt tired, my teammates would take over chest compression, which significantly improved the efficacy.

Male, 21 years, role A

We had good coordination. When one person was pressing, the others could communicate with the telecommunicator, so that the telecommunicator could adjust the instructions according to the situation timely.

Female, 42 years, role B

In addition, team-based instructions were widely recognized. Participants described the protocol as “understandable,” “precise,” and “efficient.” The beneficial effects of instruction on team cooperation were widely acknowledged.

The instructions helped us form a well-organized team, which is a process that would take a long time without the telecommunicator involved.

Female, 23 years, role C

The experts also spoke highly of the protocol. The advantages of task allocation and closed-loop communication protocols were recognized during the simulation.

The protocol is in strict compliance with the AHA guidelines and can play a good instructing role on the event of OHCA. It allows multiple lay responders to have a clear division of labor and help each other as a team, which is important for providing high-quality CPR.

However, there was little feedback on the shortcomings of the protocol, which included a high demand for the telecommunicator and the requirement for more detailed instructions during the process of switching to the chest compression implementer.

### Results of Combining Significant Quantitative Findings With Qualitative Remarks

The significant quantitative findings were integrated with qualitative remarks to reveal the participants’ classic behavioral performance that affected the quality of CPR. Performance was divided into 2 themes: procedural issues and team factors. A total of 12 items were extracted (Table 3).

**Table 3:** Participants’ behavior performance of team-based tele-instruction tool. Phase I tests were team-based cardiopulmonary resuscitation (CPR) performance and capabilities assessment with or without the team-based tele-instruction tool among individuals who have undergone single-rescuer CPR training. Phase II tests were team-based CPR performance and capabilities assessment with or without the team-based tele-instruction tool among individuals who have undergone single-rescuer and team-based CPR training. The emergency medical number in mainland China is 1-2-0.

	Behavior performance	Quantitative analysis		Qualitative analysis
		Phase I (N=20), n (%)	Phase II (N=20), n (%)	Quotes of the participants in the interview
**Procedural issues**				
	Identify cardiac arrest before making an emergency call.	18 (90)	20 (100)	—^a^
	CPR was already in progress before making an emergency call.	15 (75)	18 (90)	“My first instinct was to start first aid and then call 120.” (woman, 18, role A)
	Telecommunicator interrupted the ongoing rescue.	18 (90)	12 (60)	“I was performing 30:2 before the telecommunicator instructed me on chest compression.” (man, 18, role A)
	Caller refused or disobeyed to take CPR instructions.	5 (25)	2 (10)	—
	Caller actively gave resuscitation progress report to the telecommunicator.	3 (15)	7 (35)	“We should have told the telecommunicator that we were performing 30:2 at the beginning.” (woman, 48, role B)
	Activate the speaker function of the phone.	19 (95)	20 (100)	“I activated the speaker function of the phone to make sure my partners could hear the instructions clearly.” (man, 34, role A)
	Caller could be heard counting out compressions.	14 (70)	16 (80)	“To ensure the frequency, we counted the compression rhythm together.” (woman, 18, role C)
**Team factors**				
	The team and the telecommunicator communicated effectively.	15 (75)	18 (90)	“The instructions were clear and the responses was timely and useful.” (man, 43, role C)
	The team acted with composure and control after the telecommunicator instruction.	15 (75)	19 (95)	“I was a little flustered at the very beginning, but with the instructions of the telecommunicator, I gradually calmed down.” (woman, 20, role A)
	The team worked together to complete tasks in a timely manner.	17 (85)	20 (100)	“The telecommunicator told us exactly what we needed to do and we knew how to cooperate with our partners.” (man, 19, role B)
	The team morale was positive.	16 (80)	17 (85)	“We were all very active and involved in the work.” (man, 21, role A)
	The team monitored and reassessed the situation to the telecommunicator.	4 (20)	6 (30)	“We didn’t inform the telecommunicator of the situation until he asked.” (man, 24, role A)

^a^Not available.

## Discussion

### Overview

OHCA is a major cause of death worldwide, and high-quality CPR facilitates neurological outcomes and overall prognosis [17]. An organized multiperson resuscitation team has been reported to significantly contribute to the performance of high-quality CPR [18,19]. Nevertheless, team-based CPR requires implementers to have good communication ability, leadership, and CPR skills. Although there has been a corresponding training plan, it is still difficult for the practical rollout of team-based CPR. Telehealth technologies and services, which have become important components of the health care system [20], present a potential solution. By dialing the emergency number, telecommunicators become the first responders and are a critical link in the OHCA chain of survival, which remarkably reduces the difficulty of execution of team CPR by laypersons. However, a standardized protocol for telecommunicators to teach team-based CPR techniques through the telephone is lacking. Hence, based on the present T-CPR protocol, we creatively designed an innovative tele-instruction CPR protocol for 3 or more lay responders. Quantitative and qualitative analyses showed that the present protocol could facilitate the formation of a well-organized team and significantly improve resuscitation quality among multiple lay responders. The accuracy and effectiveness of the protocol were highly recognized by the experts and regarded as “with good compatibility and certain generalizability.”

The tele-instruction tool for multiple lay responders has good universality and promotional value. It was reported that 43.1% of OHCA resuscitation incidents involved multiple lay responders, more than half of which involved 3 or more lay responders [12]. In the case of more than 3 lay responders, team members could be shifted to accommodate the diverse needs of different circumstances. In reality, people may be reluctant to perform resuscitation because of a lack of confidence or concerns about legal complications. When resuscitation becomes a group task, the risk associated with it is shared among the individuals involved, leading to an increase in people’s willingness to participate in resuscitation efforts. Strong leadership and communication are crucial for high-quality team-based CPR, yet leadership training has not been popularized [18,21]. Our team-based tele-instruction tool effectively used the leadership role of the telecommunicator in task management. Telecommunicators who take responsibility as team leaders can guide callers to delegate various functions to other lay responders during resuscitation [22]. The promotion of the protocol will help increase people’s willingness to perform resuscitation, which is important for the promotion of health literacy in the population.

CPR has long been considered the hallmark of OHCA management, and chest compressions with an appropriate frequency and depth are a significant component of high-quality CPR. The team-based tele-instruction tool demonstrated a remarkable advantage in terms of chest compression quality. By counting loudly with a telecommunicator, the intervention group maintained a more suitable average compression rate. This was particularly obvious when the rescuers had just begun chest compressions or had just changed roles. Team-based instructions could help rescuers achieve a better effect quickly after beginning compression, shorten the time of adaptation, and facilitate efficient intrateam role alternation. In addition, the intervention group achieved a relatively greater compression depth (within 5-6 centimeters). It was assumed that team-based instructions could realize better team cooperation and more timely rotation, avoiding poor-quality resuscitation due to fatigue.

Prompt emergency response is crucial for OHCA rescue [23-25]. We found that T-CPR could improve the accuracy of OHCA identification but lengthen the response times to open the airway and first shock in both phases and the time to first chest compression, which is consistent with the findings of previous studies [26,27]. This was because of the inevitable time required for communicating with the telecommunicator and factors beyond their control [28]. However, compared with the protocol for a single lay responder, the team-based tele-instruction tool can maximize the strength of all lay responders, optimally distribute roles, and reduce interruptions in the resuscitation process. In addition, the results of this study showed that the WeCan CPR basic life support training could help shorten the delay in response. This suggests that with adequate training, the problem of response time delays in the protocol may not be as problematic. The increasing health literacy and improving resuscitation capabilities among citizens have created a favorable environment for the implementation of this protocol, opening up new possibilities for more successful resuscitation.

Feedback from the participants and experts provided support for the validity of the protocol and highlighted issues requiring improvement. First, many participants did not actively report to the telecommunicator during resuscitation, which may have wasted their valuable time. Second, telecommunicators should pay more attention to feedback from rescuers, adjust instructions in a timely manner, and offer psychological support and encouragement, which requires abundant work experience and an effective improvisation ability. However, it may be difficult for telecommunicators to achieve this on their own. Some studies used machine learning to assist telecommunicators in OHCA recognition and achieved better results [29,30]. Integrating a well-established team-based tele-instruction protocol into telehealth technologies and service systems may significantly enhance operator proficiency and improve OHCA management.

Nevertheless, this study had some limitations. First, a simulation trial was conducted with people who had mastered single-rescuer CPR skills to perform a targeted evaluation of the tool. However, in real-life OHCA situations, it is not certain whether lay responders equipped with resuscitation skills would be recruited. Therefore, we will further expand the compatibility by collecting more representative data to demonstrate the efficacy of the protocol. Second, due to some practical barriers, such as the disconnection of the call and the time-consuming task of information registration, our test may not have been completely simulated. Finally, although the study design could reduce the effect of confounding factors between the groups, the practice effect did exist and could have led to motivational bias.

### Conclusions

In this study, we developed an innovative team-based tele-instruction tool and assessed its effectiveness in ensuring the delivery of high-quality CPR. This tool facilitated the efficient organization of resuscitation teams, promoted effective cooperation, and enhanced compression quality among multiple lay responders. Consequently, we propose that the efficacy and applicability of the team-based tele-instruction tool should be evaluated in the telehealth emergency service systems to enhance resuscitation outcomes in real-life rescue scenarios.

## Abbreviations

AED: automated external defibrillator
AHA: American Heart Association
CCF: chest compression fraction
CPR: cardiopulmonary resuscitation
OHCA: out-of-hospital cardiac arrests
T-CPR: telephone-assisted cardiopulmonary resuscitation
